# Morphometric Variations in the Grasshopper, *Chromacris speciosa* from Two Localities of Pernambuco in Northeastern Brazil

**DOI:** 10.1673/031.012.7901

**Published:** 2012-07-09

**Authors:** Roberta Araújo Cisneiros, Argus Vasconcelos de Almeida, Gabriel Rivas de Melo, Cláudio Augusto Gomes da Câmara

**Affiliations:** ^1^ Universidade Federal Rural de Pernambuco, Departamento de Biologia, Av. D.Manoel de Medeiros, s/n, Dois Irmãos, Recife, PE, CEP 52171-900.; ^2^ Universidade Federal Rural de Pernambuco, Departamento de Biologia, Av. D.Manoel de Medeiros, s/n, Dois Irmãos, Recife, PE, CEP 52171-900.; ^3^ Universidade Federal Rural de Pernambuco, Departamento de Estatistica e Informática, Av. D.Manoel de Medeiros, s/n, Dois Irmãos, Recife, PE, CEP 52171-900.; ^4^ Universidade Federal Rural de Pernambuco, Programa de Pós-graduação em Entomologia Agrícola, Laboratório de Produtos Naturais Bioativos, Rua Dom Manoel de Medeiros, s/n, Dois Irmãos, Recife, PE, CEP 52171-900.

**Keywords:** atlantic rainforest fragment, grasshopper soldier, populations

## Abstract

The present study describes morphometric variations in the grasshopper, *Chromacris speciosa* (Thunberg, 1824) (Orthoptera: Acridoidea: Romaleidae) from two locations in the state of Pernambuco, Brazil. The distance between the sites chosen for collections (Recife and São Lourenço da Mata) is approximately 16 km. The investigation was based on a comparative study of external morphological characteristics of the grasshoppers. Morphometric measurements took into account the different body parts and appendages. Statistical analysis of the measurements revealed significant differences in the size of the specimens between the two locations. Homogeneity tests of the covariance and equality matrices between mean vectors of the results revealed that the grasshopper populations in Recife and São Lourenço da Mata are distinctly different. These findings provide morphological evidence for intraspecific variation in morphological characteristics of the *C. speciosa* populations from the two locations.

## Resumo

Este trabalho descreve variações morfométricas em *Chromacris speciosa* (Thunberg, 1824) (Orthoptera: Acridoidea: Romaleidae) de duas localidades de Pernambuco - Brasil. A distância entre as duas localidades que foram escolhidas com sítios de coleta (Recife e São Lourenço da Mata) é cerca de 16 Km. A investigação deste fenômeno fundamentou-se no estudo comparativo de caracteres morfológicos externos dos gafanhotos. As medições morfométricas levaram em consideração as diferentes partes do corpo e apêndices dos gafanhotos. A análise estatística das medições dos caracteres morfológicos externos dos gafanhotos mostrou diferenças significativas no tamanho dos espécimes de ambas as localidades. Os testes de homogeneidade das matrizes de covariância e os de igualdade entre os vetores de média dos resultados obtidos mostraram que as populaçães de gafanhotos em Recife e São Lourenço da Mata são distintas. Esses resultados sugerem que as evidências morfométricas revelam uma variação intra-específica dos caracteres morfológicos das populações de *C. speciosa* de das duas localidades.

## Introduction

The systematics of grasshoppers in the neotropics is currently in a state of flux, and, although considerable information has been added in recent years, knowledge on grasshoppers remains insufficient. Species from entire regions are either little understood or completely unknown. This is particularly the case in northeastern Brazil, where records of acrididae are very rare (Duranton et al. 1987).

Acridology has recently been recognized as an important area of study, due to the economic importance of the devastation produced by locusts. Acrididae may be either gregarious and migratory, or solitary and sedentary, but any solitary species may transform into a gregarious or migratory species (Liebermann 1935).

The superfamily Acridoidea, which is found in the neotropics, is divided into eight families. Romaleidae is only found in the Americas, with *Chromacris speciosa* distributed throughout eastern Brazil, Paraguay, Uraguay and Argentina (Duranton 1987). According to Roberts & Carbonell (1982), its occurrence in South America is restricted to Ecuador, Peru, Bolivia, Paraguay, Colombia, Venezuela, and Argentina. *C. speciosa* is popularly known as the “soldier grasshopper,” and is characterized by its medium size, generally dark green color, and orange-red hind wings. Its main habitat is a bush environment with vegetation principally consisting of species in the families Solanaceae, Myrtaceae, and Gramineae (Duranton et al. 1987). *C. speciosa* is found in all regions of Brazil, especially in the states of Amapá and Para in the northern region; Ceará, Pernambuco, and Bahia in the northeastern region; Mato Grosso do Sul and Goiás in the central western region; Minas Gerais, Rio de Janeiro and São Paulo in the southeastern region; and Santa Catarina and Rio Grande do Sul in the southern region (Silva et al. 1968; Duranton 1987).

The soldier grasshopper attacks plants from different families, including Fabaceae, Solanaceae, Myrtaceae, Poaceae, and Gramineae. In observations made by Silva et al. (1968), it was found feeding on leaves of the mesquite bean (*Prosopis juliflora*, Fabaceae), pigeon pea (*Cajanus cajan*, Fabaceae), eggplant (*Solanum melongena*, Solanaceae), jurubeba (*Solanum paniculatum*, Solanaceaey, tomato (*Lycopersicon esculentum*, Solanaceae), and *Eucalyptus* sp. (Myrtaceae). There are also records of *C. speciosa* attacks on sugarcane (*Saccharum officinarum* Poaceae) in northeastern Brazil (Guagliumi 1973; Roberts 1982), and on rice (*Oryza poaceae*, Poaceae) in Peru, causing significant damage to crops.

According to Liebermann (1935), *C. speciosa* may be easily mistaken for *Chromacris. miles* (Drury), as the differences between the two are minimal. However, the former is a southern hemisphere species, while the latter lives in Central America and the northern most portion of the southern hemisphere. Recently, Almeida & Câmara (2008) reported the occurrence of young forms of *C. speciosa* in various types of environments in the Estação Ecológica de Tapacurá (Ecological Station of Tapacurá), highlighting the occurrence in human-influenced environments, but also recording the occurrence in boundary vegetation of the forest.

According to Mayr (1969), the phenotype of animal populations from a single species often varies with the location, season of the year, and type of habitat. Therefore, differences between populations of phenotypically similar specimens may reflect an intraspecific difference, or a variation in the species under study. However, comparing *C. speciosa* specimens that occurred in three locations in South America (Carabobo, Venezuela; Santa Cruz, Bolívia; Corup´, Brazil), Roberts & Carbonell (1985) found that, even with the considerable evidence regarding the geographical differences between the locations in South America in which the species were found, it was not possible to recognize subspecific elements for *C. speciosa*.

In another study, Duranton et al. (1982) used the morphometric methods described by Dirsh to compare specimens from a single population, studying 50 individuals of each sex. Which is the recommended number for such studies even when the population is morphologically homogeneous. Bruner (1911; 1913; 1919) and Liebermann (1935) described new grasshopper species, but did not cite the number of individuals used for the morphometry. A number of other studies have reported the use of morphometry on Acridoidea for the description of new species using a low number of specimens (Bruner 1907; Rehn 1909, 1913; Hebard 1924; Piza Júnior 1953; Blackith & Albrecht 1960, Descamps 1977; Amédégnato & Descamps 1978, 1979). Using Dirsh's method regarding the morphological structures used for measurement, Uvarov (1966) carried out a study on polymorphism in the gregarious and solitary phase in a particular species of grasshopper, while also using Dirsh's method to characterize species.

Studying the morphology of *C. speciosa* in four different locations in Argentina, Turk & Barrera (1976) determined differences between individuals from different regions.

Other studies have been carried out using morphometry to determine the existence of a polymorphic phase in the species *Austracris guttulosa* (Walker), which occurs in Australia (Elder 1996), as well as the speciation process of African grasshoppers from the genus *Afrophlaeoba* (Hochkirch 2005). Morphometric studies help characterize phases (polymorphism), aid in studying mixtures of two populations of different origins in a single place, and give evidence of geographic advantages between populations.

Morphometrics can continue to play a vital role in evolutionary studies of development as its results generate questions both for its practioners and other biologists to explore (Roth & Mercer 2000).

There are currently no published reports of morphological variations in two populations of *C. speciosa* from two different Mata Atlantic rainforest regions of northeastern Brazil. Our study examines morphometric variations of *C. speciosa* specimens from the Universidade Federal Rural de Pernambuco (UFRPE) campus in Recife, and Tapacurá Ecological Station in São Lourenço da Mata (EET), both located in the state of Pernambuco.

## Materials and Methods

This study was carried out through observation and collection expeditions of the specimens of *C. speciosa* that live on the campus of the UFRP and at the EET. The former, even though it is quite modified by anthropoligical actions, showed a great variety of vegetation types because of physiognomic aspects, rainfall indices, and soil depth. The EET had two remnants of Atlantic Forest, known as the camocim and toró, and divided by the Tapacurá dam reservoir, with average annual rainfall of 1300 mm, and seasonal semi-deciduous type forest on seasonally dry lowland

*C. speciosa* was identified through direct comparison with previously identified specimens from the entomology collection of the Biology Department of the UFRPE. The adult specimens of *C. speciosa* were collected with entomological nets, predominately on jurubeba (*S. paniculatum*) as a host plant. A total of 307 specimens of *C. speciosa* were collected, of which 145 were from Recife (70 males and 75 females), and 162 were collected from Säo Lourenço da Mata (63 males and 99 females). The specimens were collected using entomologic nets. Male and female specimens were carefully separated, labeled, and preserved in 70° alcohol.

**Figure 1.**
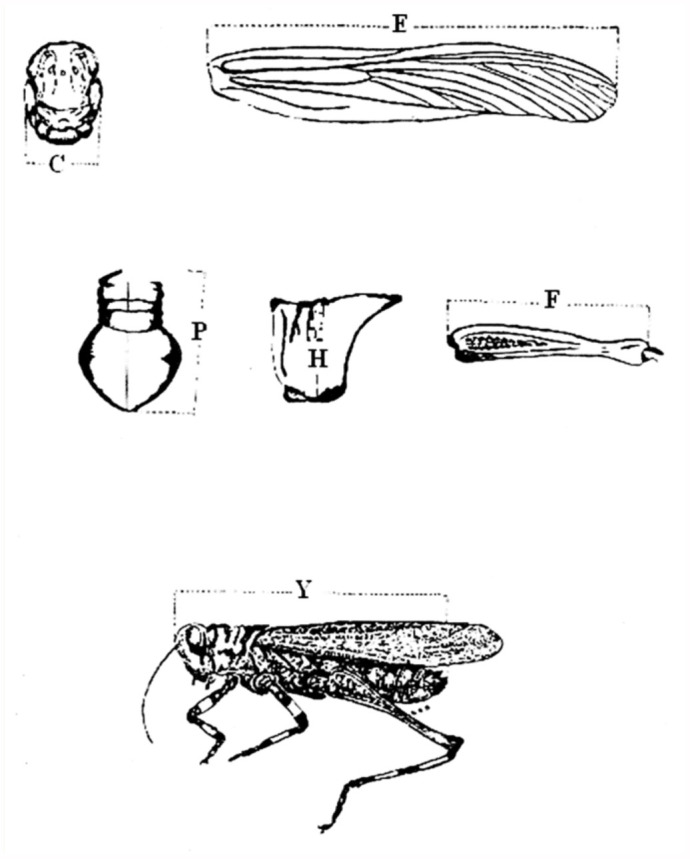
Morphometric conventions used on *Chromacris speciosa*. C = maximum head width (cheek to cheek); E = wing length (from axial region to apex); P = pronotum length; F = total femur length of metathoracic leg; Y = total body length (from front of head to terminal region of abdomen); H = pronotum height. High quality figures are available online.

### Morphometric studies

Morphometric analyses were performed at the entomology laboratory of the UFRPE Biology Department. Methodology used in this experiment was according to the methods described by Uvarov (1966). The obtained morphological measurements data were submitted to a multivariate analysis adopted by Anderson (1958) and Johnson and Wichern (1989). The morphologic structures were measured using a Mitutoyo pachymeter (0.02 mm). Figure 1 illustrates the conventions adopted for morphological structure measurements of *C. speciosa*.

The following statistical parameters were analyzed based on the morphometric measurements of grasshoppers from the different collection sites: mean, standard deviation, coefficient of variation, and frequency distribution. To study differences between the sets of morphometric measurements of the soldier grasshoppers from the different collection sites, the following multivariate analysis methods were employed: equality test of two covariance matrices, equality test of two mean vectors, and linear discriminant function. The first two methods were employed to determine the expression quality of the grasshopper measurements in the two locations, that is, to objectively test the following hypotheses: H_0_ — there is no differentiation between the sets of morphometric measurements of the grasshoppers from the two locations (test hypothesis), and H_1_ — there is differentiation between the sets of morphometric measurements of the grasshoppers from the two locations (alternative hypothesis). The third method was adopted as a complementary measure to analytically construct a location classification based on the morphometric measurements of the grasshoppers. This method is only applicable if the H1 hypothesis is accepted (Anderson 1958; Johnson & Wicher 1989).

## Results and Discussion

On the UFRPE campus and at the EET, grasshoppers were found attacking solanaceae, especially the jurubeba (*S. paniculatum*), the leaves of which had undergone considerable damage. Through field observations, accentuated differences in overall size of the grasshoppers were noticed between the two locations. Regarding the measurements, certain difficulties occurred in the proper placement of the pachymeter over the morphometric structures, but a technique that adequately avoided slippage was employed, thereby minimizing the margin of error.

The structures measured (Figure 1) and the position in which the arms of the pachymeter were placed are as follows: a) Maximum head width (C) — to obtain the maximum width of the head, the arms of the pachymeter were placed in the cheek region ventrally, and not over the eyes, in order to offer greater firmness to the instrument, and because this region was the widest; b) Wing length (E) — the measurement was taken by placing one arm of the pachymeter on the axial region, and the other on the apical region of the wing (due to the frequent handling, the wings occasionally became detached from the body of the insect, making it necessary to place them on a flat surface in order to take the measurements); c) Pronotum length (P) — the measurement was taken from the dorsal portion in the metazone region, from the prescutum to the extension of the postscutellum; Pronotum height (H) — this measurement was taken by placing the arms of the pachymeter on the scutellum region, because this region was the highest; d) Total femur length of the metathoracic leg (F) — this measurement was taken on the dorsal part of the femur in order to offer greater firmness to the pachymeter, with one arm placed immediately after the trochanter, and the other placed at the beginning of the femur-tibia joint; e) Total body length (Y) — the arms of the pachymeter were placed at the front of the head, and at the terminal region of the abdomen, with the specimens placed on a flat surface. Table 1 displays the results of these conventional measurements of the different body parts and appendages of *C. speciosa*.

Regarding the identification of the specimens in the collections, *C. speciosa* is often confused with *C. miles*, due to the morphological similarities between the two. However, besides the fact that *C. miles* does not occur in the regions from which the samples were taken, it has distinct morphological details, such as the yellow posterior margin of the pronotum that is uninterrupted by spots, and the green coloration of the thigh joint rings on the mesothoracic and metathoracic legs. In contrast, *C. speciosa* has a yellow posterior margin of the pronotum that is interrupted by either black or green spots, and thigh joint rings on the mesothoracic and metathoracic legs either entirely or partially of a yellow coloration.

The characterization of the samples was initially performed through three descriptive measurements of the morphometry of the grasshoppers from the EET and the UFRPE campus: the coefficient of variation, mean, and standard deviations. Table 1 displays the respective values of these measures.

Comparing the morphometric measurements of the grasshoppers from the UFRPE campus to those from the EET, it is evident that the total femur length of the metathoracic leg, the maximum head width (cheek to cheek), the wing length (axial region to apex), the pronotum length, and the total body length (front of the head to the terminal region of the abdomen) of the grasshoppers from the UFRPE campus were smaller than those from the EET. Although the grasshoppers from the EET were larger in nearly all the measurements, those from the UFRPE campus had a greater pronotum height. The results of the grasshoppers from the UFRPE campus with regard to standard deviation are greater that those obtained from the grasshoppers at the EET.

The results of the coefficient of variation for the grasshoppers from the UFRPE campus are higher than those obtained at the EET. Therefore, in terms of variability of the specimens collected on the UFRPE campus, the following measurements were more heterogeneous: femur length, maximum head width, wing length, pronotum length, total length, and pronotum height. The means of the measurements of total length, pronotum length, wing length, and maximum head width were greater on the females from the EET, whereas the means of the measurements of pronotum height and femur length were greater on the females from the UFRPE campus. Analyzing the thoracic appendages, the females from the EET had larger forewings, but shorter hind legs, whereas those from the UFRPE campus had smaller forewings and longer hind legs.

The pronotum on females collected from the UFRPE campus had greater height and lesser length than that of the females from the EET. Thus, the present study suggests that the pronotum is proportional to total length, as the females from the UFRPE campus were smaller and had a smaller pronotum length than the females from the EET. With regard to head dimensions, the females from the EET had a wider head than those from the UFRPE campus. Based on the values from the coefficient of variation of femur length, wing length, and pronotum height, the females from the EET had greater heterogeneity, whereas greater heterogeneity in the measurements obtained for maximum head width, total length, and total pronotum length occurred in the females from the UFRPE campus.

**Table 1.**
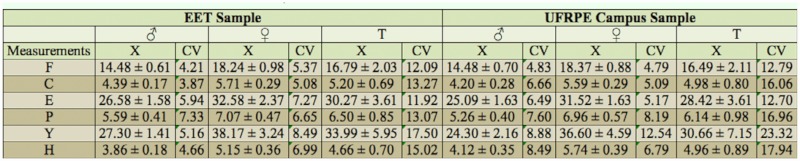
Morphometric measurements (mm) of grasshoppers (*Chromacris speciosa*) from the EET and UFRPE locations. F = femur length; C = maximum head width; E = wing length; P = pronotum length; Y = total length; H =pronotum height; X = mean; CV = coefficient of variation; T = morphometric measurements of males and females combine.

**Table 2.**
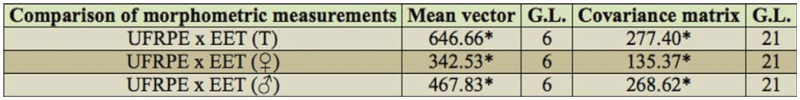
Values of multivariate tests (chi-square) for mean vectors and covariance matrix of the morphometric measurements of *Chromacris speciosa* from the UFRPE and EET locations. * significant at 1%

With regard to the thoracic appendages, males from the EET had larger wings than those from the UFRPE campus, although both had hind legs of the same size. The pronotum on males from the UFRPE campus had greater height and lesser length, whereas the pronotum on the males from the EET had lesser height and greater length. Thus, although the males from the UFRPE campus had shorter pronotum lengths, they are taller that those at from the EET, which had greater pronotum length, but were shorter in height. Like in females, pronotum length was proportional to total length in males. The head was wider on males from the EET. The results of the coefficient of variance for males from the UFRPE were higher than those from the EET, implying greater heterogeneity among the males from the UFRPE campus.

Turk & Barrera (1976) performed a morphometric study on the same species from four collection sites in Argentina (Tapia, Benjamin Paz, San Miguel de Tucumán, and Mista), with emphasis given to the measurements of total length, maximum head width, femur length, and wing length. Comparing the results from male and female grasshoppers from Argentina and those obtained in the present study, the grasshoppers from both collection sites in Pernambuco had greater mean measurements of wing length and head width, as well as lesser total length and femur length. Therefore, the grasshoppers of the present study are smaller than those from Argentina.

To determine whether the morphometric differences between the grasshoppers at the UFRPE, EET and Argentine locations are real, males and females were considered separately for analysis purposes. The results revealed that the males and females from the UFRPE and EET are smaller, with underdeveloped hind legs, larger hind wings, and a wider head. Comparing these measurements with those obtained for males in different locations in Argentina, the grasshoppers from the EET and UFRPE exhibited greater values for these measurements than those from Argentina. However, femur length was greater in male grasshoppers from Argentina.

With the objective of studying differences between the set of morphometric measurements of *C. speciosa* at the EET and UFRPE locations rather than isolated morphometric characteristics, an overall study was carried out such that all the measurements were considered in the multivariate analysis in order to determine possible differences that could be expressed in the grasshopper as a whole between one location and another. Equality of covariance matrices and mean vectors were employed to identify differentiation, or lack thereof, between the set of morphometric measurements of the grasshopper under study from the UFRPE campus the EET. Table 2 displays the values obtained in the multivariate analysis of the morphometric measurements of the grasshoppers collected from the two sites.

**Table 3.**
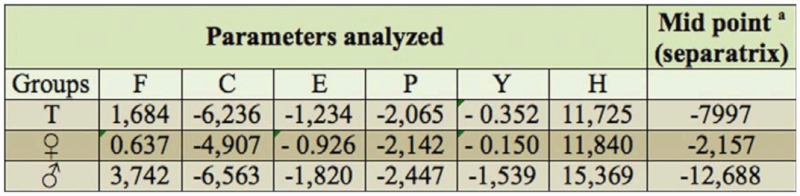
Discriminant function of morphometric measurements of *Chromacris* speciosa from the UFRPE and EET locations. ^a^ Note: if the discriminant value for a certain value of morphometric measurements (6 measurements) is greater than the midpoint, the vector is from the UFRPE location, otherwise it is from the EET location. ^b^ T = morphometric measurements for males and females combine.

The results revealed an overview of the set of measurements. The males and females from the EET were compared both together and separately with those from the UFRPE by the comparative tests of means and covariance matrices, revealing significant differences in the grasshoppers from the two locations (Table 2). The results of both tests suggest intraspecific variation, as there are morphometric differences between the *C. speciosa* specimens collected from the two sites.

With intraspecific variation determined, an additional multivariate analysis was performed for the construction of an allocation function. With this analysis, it was possible to identify the collection site of a given grasshopper with a 99% degree of reliability. From the morphometric measurements, it was possible to obtain three discriminant functions: The first, for males and females together, was denominated general discriminant function (T); the second, for males (♀), and the third, for females, (♂), were both denominated specific discriminant functions. Table 3 displays the data obtained for the three discriminant functions using the morphometric measurements of the grasshoppers from the UFRPE and EET.

Choosing a male or female grasshopper at random from one of the two collection sites, its origin could be established through the application of its morphometric measurements using the general or specific discriminant functions. For example, let the measurements (in millimeters) of a male grasshopper be 14.8 for femur length, 4.70 for maximum head width, 26.8 for wing length, 6.00 for pronotum length, 4.20 for pronotum height, and 28.9 for total length. Applying these measurements to the following discriminant population equation:

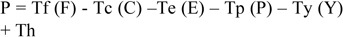

for which P = EET or UFRPE population; Tf, Tc, Te, Tp, Ty, and Th are, respectively, femur length, maximum head width, wing length, pronotum length, total length, and pronotum height for males and females combined; and F, C, P, Y, and H are the morphometric measures obtained. Substituting the values in the equation, we have the following:
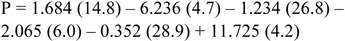

Thus, the discriminant value obtained from the male grasshopper in question is 10.775. As this value is greater than that obtained for the separatrix mid point (Table 3), it can be concluded that this specimen pertains to the UFRPE campus location.

The results reported in the present study regarding the analyses of morphometric measurements taken for grasshopper populations from two locations in the state of Pernambuco (Brazil) revealed significant evidence of differentiation between the EET and UFRPE locations, thereby suggesting morphometric evidence revealing intraspecific variation of *C. speciosa* associated to the two locations of origin.
